# Supplementary material for: KDR (VEGFR2) Genetic Variants and Serum Levels in Patients with Rheumatoid Arthritis
Source: Biomolecules. 2019 Aug 9;9(8):355. doi: 10.3390/biom9080355 (PMC6727087; doi:10.3390/biom9080355)
Supplement: Supplementary file 1 [file biomolecules-09-00355-s001.pdf]

Table S1. SNPs information and genotyping results for RA patients and control group.

SNP ID	Allele	SNP Type/ Amino Acid Change	MAF		
			RA	Control	HapMap-CEU
**rs1870377**	+1416A/T	exon 11 H472Q	0.32	0.27	0.21
**rs2305948**	+889 G/A	exon 7 V297I	0.18	0.14	0.15
**rs2071559**	-604 C/T	promoter region	0.48	0.60	0.50

MAF - minor allele frequency; CEU - Utah residents of northern and western European ancestry; UTR – untranslated region

Table S2. Association between genotypes of KDR+1416A/T and clinical characteristics among RA patients.

Parameter	AA		AT+TT		p*
	N	median (IQR)	*N*	median (IQR)	
Age [years]	447	50 (32 - 60)	448	51.5 (34.5 – 60)	0.612
Disease duration [years]	239	10 (5 - 16)	267	9 (4 – 15)	0.116
Larsen	255	3 (3 -4)	282	3 (2 - 3)	0.334
Number of tender joints	152	7 (2 – 11)	163	8 (4 -13)	0.130
Number of swollen joints	152	3 (1 – 7)	165	3 (1 – 8)	0.217
ESR [mm/h]	251	30 (17 – 51)	284	30 (16 – 50)	0.586
CRP [mg/L]	153	14 (6 – 32)	169	11.7 (5.3 – 32)	0.329
Hemoglobin [g/dL]	154	12.6 (11.7 – 13.4)	168	12.8 (11.7 – 13.7)	0.604
VAS [mm]	147	51 (30 – 71)	161	53 (34 – 70)	0.575
DAS 28-CRP	147	5 (3.8 – 5.7)	164	5 (3.8 – 5.9)	0.454
HAQ	129	1.62 (1 – 2)	136	1.5 (1 – 2)	0.398
PLT [x10^3/mm^3]	153	305 (290 – 350)	168	308 (251 – 369)	0.911
Creatinine	153	0.7 (06 – 0.8)	166	0.7 (06 – 0.8)	0.481
					p**
	N	n (%)	*N*	n (%)	
Women	*450*	349 (82%)	*448*	369 (78%)	**0.07**
RF presence	*251*	158 (63%)	*283*	203 (72%)	**0.03**
anti-CCP presence	*154*	116 (75%)	*171*	141 (82%)	0.114

Table S3. Association between genotypes of KDR -604 C/T and clinical characteristics among RA patients.

Parameter	TT		TC+CC		p*
	N	median (IQR)	*N*	median (IQR)	
Age [years]	175	55 (48 – 63)	777	48 (30 – 59)	0.679
Disease duration [years]	135	10 (5 - 16)	388	10 (4.5 – 15.5)	0.748
Larsen	145	3 (3 – 4)	420	3 (2 – 4)	0.241
Number of tender joints	81	8 (3 – 13)	256	7 (3 – 11)	0.466
Number of swollen joints	83	4 (1 - 7)	256	3 (1 – 7)	0.483
ESR [mm/h]	144	31 (18 - 50)	419	30 (16 – 50)	0.728
CRP [mg/L]	83	18 (7 - 40)	260	12 (6 – 30)	0.066
Hemoglobin [g/dL]	84	12.3 (11.4 – 13.2)	259	12.8 (11.8 – 13.6)	0.122
VAS [mm]	79	55 (40 – 72)	251	52 (32 – 71)	0.525
DAS 28-CRP	80	5.6 (4.1 – 6.2)	253	5 (3.9 – 6.7)	0.138
HAQ	71	1.5 (1 – 2)	215	1.5 (1 – 2)	0.670
PLT [x10^3/mm^3]	84	310 (260 – 375)	258	305 (245 – 381)	0.605
Creatinine	82	0.7 (0.6 – 0.8)	258	0.7 (0.6 – 0.8)	0.977
					p**
	N	n (%)	*N*	n (%)	
Women	*176*	149 (85%)	*779*	607 (88%)	**0.046**
RF presence	*144*	99 (69%)	*415*	286 (69%)	0.970
anti-CCP presence	*84*	65 (77%)	*262*	213 (81%)	0.431
